# Post-translational Modifications of the CARMA1-BCL10-MALT1 Complex in Lymphocytes and Activated B-Cell Like Subtype of Diffuse Large B-Cell Lymphoma

**DOI:** 10.3389/fonc.2018.00498

**Published:** 2018-11-09

**Authors:** An Thys, Tiphaine Douanne, Nicolas Bidère

**Affiliations:** Team SOAP, CRCINA, Institut National de la Santé et de la Recherche Médicale, CNRS, Université de Nantes, Université d'Angers, Nantes, France

**Keywords:** CARMA1, CARD11, BCL10, MALT1, CBM, post-translational modifications, diffuse large B-cell lymphoma, ABC DLBCL

## Abstract

Piracy of the NF-κB transcription factors signaling pathway, to sustain its activity, is a mechanism often deployed in B-cell lymphoma to promote unlimited growth and survival. The aggressive activated B-cell like (ABC) subtype of diffuse large B-cell lymphoma (DLBCL) exploits a multi-protein complex of CARMA1, BCL10, and MALT1 (CBM complex), which normally conveys NF-κB signaling upon antigen receptors engagement. Once assembled, the CBM also unleashes MALT1 protease activity to finely tune the immune response. As a result, ABC DLBCL tumors develop a profound addiction to NF-κB and to MALT1 enzyme, leaving open a breach for therapeutics. However, the pleiotropic nature of NF-κB jeopardizes the success of its targeting and urges us to develop new strategies. In this review, we discuss how post-translational modifications, such as phosphorylation and ubiquitination of the CBM components, as well as, MALT1 proteolytic activity, shape the CBM activity in lymphocytes and ABC DLBCL, and may provide new avenues to restore vulnerability in lymphoma.

## Introduction

Diffuse large B-cell lymphoma (DLBCL) is the most prevalent aggressive B-cell non-Hodgkin lymphoma (NHL) and accounts for 30–40% of diagnosed NHL (1, 2). The current standard treatment, called R-CHOP, consists of a combination of chemotherapies including cyclophosphamide, doxorubicin hydrochloride, vincristine and prednisone, supplemented with the CD20 antibody rituximab. Patient 3-years progression free survival upon R-CHOP treatment ranges from 30 to 70%, indicating that although all DLBCL are characterized by dense neoplastic B-blasts, the DLBCL group represents many different entities with diverse clinical outcomes (1, 2). Gene expression profiling studies from the early 2000's identified two major subtypes of DLBCL, i.e., germinal center B-cell like (GCB)-DLBCL and activated B-cell like (ABC)-DLBCL (3–6). GCB-DLBCL generally express genes that can be detected in germinal center B-cells and are thought to be derived from centroblasts, whereas ABC DLBCL's expression profile is closer to mature plasma cells and are assumed to originate from plasmablastic cells prior to germinal center exit (3, 7, 8). The importance of the ABC/GCB DLBCL classification has been acknowledged by the World Health Organization and the European Society for Medical Oncology, as clinical response to R-CHOP are divergent between subtypes, with ABC DLBCL having a significantly inferior response to treatment (1, 4, 9). However, gene expression profiling is an expensive method not customarily available in routine pathology laboratories and has universal standardized criteria for its use. Furthermore, determining cell of origin by the classical immunohistochemistry method has led to contradictory results (9).

**Figure 1:**
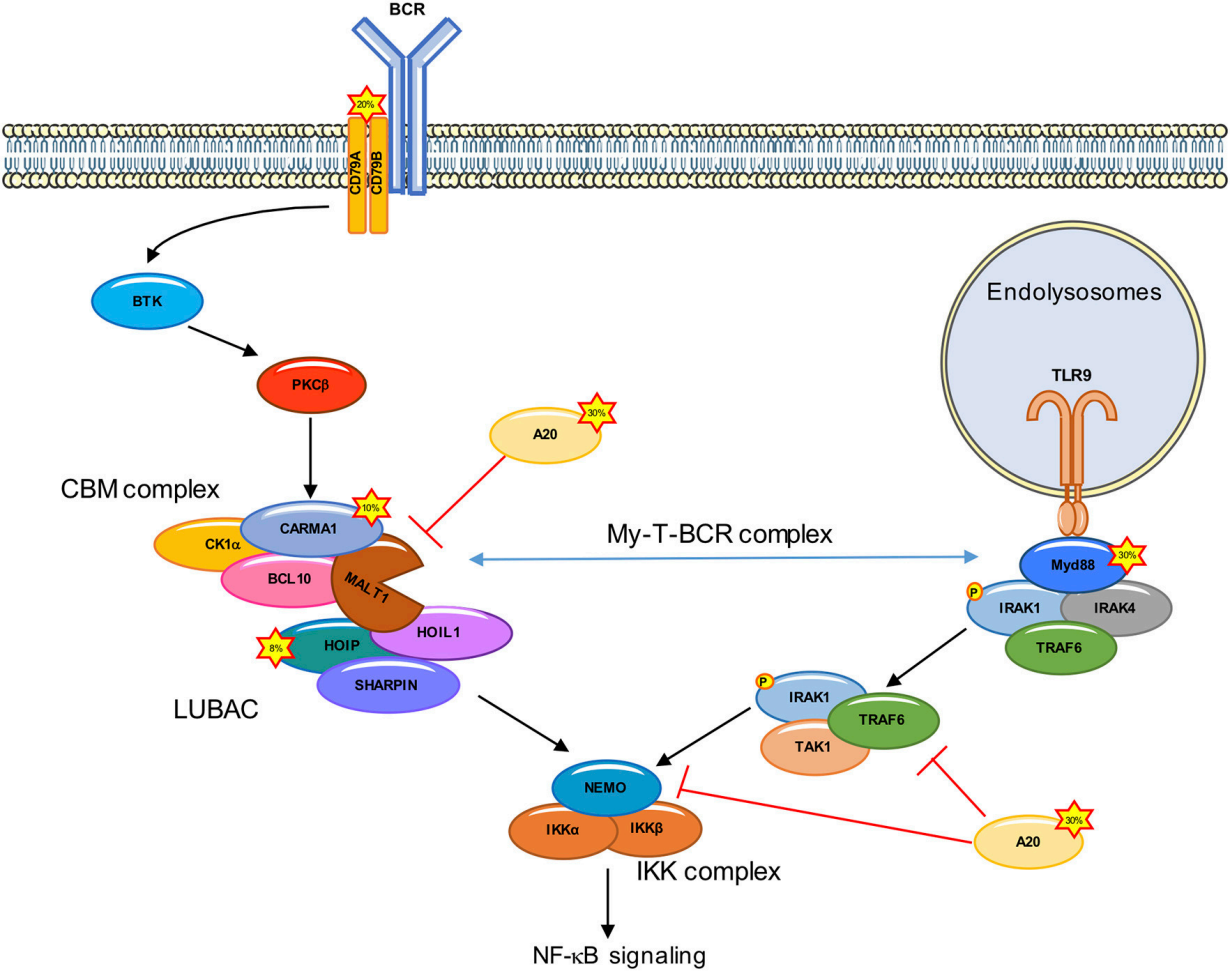
Deregulated NF-κB signaling in ABC DLBCL. In ABC DLBCL, a combination of genetic lesions leads to an effective hijacking of the BCR signaling cascade, which culminates with the assembly of the CARMA1-BCL10-MALT1 (CBM) complex, and the subsequent IKK/NF-κB activation. Mutations in ~20% of ABC DLBCL cases occur in the upstream CD79A/B. CARMA1 is mutated in ~10% of ABC DLBCL, leading to a constitutive recruitment of BCL10 and MALT1. Rare polymorphisms in the gene encoding for the LUBAC subunit HOIP (~8% of cases) increase the recruitment and activation of the IKK complex. Approximately 30% of ABC DLBCL have A20 loss of function. The Toll-like receptor adaptor MYD88 is also mutated in ~30% of ABC DLBCL. ABC DLBCL carrying both CD79A/B and MYD88 mutations form a signalosome at the endolysomome, consisting of the MYD88, TLR9, and BCR (My-T-BCR complex) which recruits the CBM.

The gene expression profile of ABC DLBCL is highly similar to that of normal B cells upon stimulation of their antigen receptors (3). Notably, nearly all cases of ABC DLBCL are characterized by a constitutive activation of the NF-κB transcription factors as a driver of lymphomagenesis (10, 11). The NF-κB heterodimers of Rel-family proteins are normally tethered to their cognate inhibitors, IκBs in the cytosol of cells. The ligation of the B-cell receptor (BCR) engages the signaling cascade of Burton's tyrosine kinase (BTK), phospholipase Cγ, which culminates in the activation of the protein kinase Cβ (PKCβ). This results in the assembly of a multi-protein complex that contains the scaffold Caspase recruitment domain family member 11 (CARD11, also called CARMA1), the adaptor B cell CLL/lymphoma-10 (BCL10), and the MALT1 paracaspase (MALT1) (CARMA1-BCL10-MALT1, CBM complex). The CBM organizes as a filamentous high-order structure that serves as a docking surface for the recruitment of signaling adaptors and of the inhibitor of NF-κB kinase (IKK), composed of the catalytic subunits IKKα and IKKβ, plus the regulatory subunit IKKγ (also known as NEMO) (12). IKK subsequently phosphorylates and thereby marks IκBs for proteasomal degradation (13–16) (Figure 1). IκB-free NF-κB translocates to the nucleus and initiates the transcription of specific target genes involved in inflammation, immune regulation, cell proliferation and apoptotic prevention (17). As a result, these lymphoma cells develop a profound addiction to NF-κB, opening up new opportunities for therapeutic treatments (18).

In 2006, the Staudt laboratory engineered genomic-scale interference screens to search for genes required for the survival of ABC DLBCL-derived cell lines, and found that silencing the CBM core subunits was toxic (19). Further supporting a crucial role for this complex, gain-of-function mutations in the CARD11 gene encoding for CARMA1 were found in ~10% of ABC DLBCL tumors (11, 16). Those mutations were mapped to the CARMA1 coiled-coil domain, a region which normally locks the protein in an inactive conformation unless phosphorylated by PKCβ (20). CARMA1 mutants are constitutively activated, and form high-order signaling complexes (16). Nevertheless, the prevalence of CARD11 mutations was not sufficient to explain the addiction of all ABC DLBCL cell lines to the CBM complex (19). This was resolved with the discovery of mutations in the immune receptor tyrosine-based activation motive of CD79A or CD79B in 20% of ABC DLBCL, that lead to an effective hijacking of the downstream BCR signaling (13).

In addition to CD79A/CD79B and CARD11 mutations, ~40% of ABC DLBCL cases harbor a gain-of-function mutations (L265P, V217F, S219C, M232T, S243N, T294P amino acid substitution) in Myeloid differentiation primary response 88 (MYD88) (21). L265P is the most common MYD88 mutation found in ABC DLBCL (29%), while it is rare in the GCB subtype (21). This mutation enables the spontaneous assembly of IL-receptor-associated kinase-1 (IRAK1) and IRAK4, allowing IRAK4 to phosphorylate IRAK, which promotes the oligomerization and activation of the E3 ligase TNF receptor associated factor 6 (TRAF6). TRAF6 recruits TGFβ activated kinase1 (TAK1) and MAP3K7 binding protein 2 (TAB2) (22–24). TRAF6 then ubiquitinates TAK1 and NEMO. Ubiquitination of NEMO leads to a stabilization of the IKK complex, while the ubiquitination of TAK1 activates its kinase activity and leads to the phosphorylation of IKKα and downstream NF-κB activation (25, 26) (Figure 1). A clinical trial targeting ABC DLBCL with the BTK inhibitor Ibrutinib led to a response rate of 80% in ABC DLBCL carrying both CD79A/CD79B and MYD88 mutations (27). Recently, Phelan et al. explain this increased sensitivity by linking BCR and MyD88 signaling through the description of a new multiprotein supercomplex formed by MYD88, TLR9, and the BCR (My-T-BCR). This complex, located at the endolysosomes, recruits the CBM complex and drives NF-κB signaling in Ibrutinib-responsive cell lines and biopsies (28) (Figure 1). Nevertheless, the overall responsive rate to Ibrutinib in ABC DLBCL is only 37% (21), and this can partly be explained by mutations downstream of BCR or MYD88 (16, 29–33).

The CBM complex orchestrates a choreography of post-translational modifications. This signalosome forms a scaffolding platform for the recruitment of a variety of kinases and phosphatases, E3 ligases and deubiquitinating enzymes (DUBs), and unleashes MALT1 protease. A case study is provided with the linear ubiquitin assembly complex (LUBAC). This ternary complex consists of the E3 ligases HOIL1-interacting protein (HOIP) and RanBP-type and C3HC4-type zinc finger-containing protein 1 (HOIL1), together with the adaptor and SHANK-associated RH domain interactor (SHARPIN). The LUBAC binds the CBM to authorize the recruitment and phosphorylation of IKK in response to the engagement of antigen receptors, as well as in ABC DLBCL by conjugating linear chains to substrates (34–38). In addition, the LUBAC subunit HOIL1 is specifically cleaved by MALT1 to promote full NF-κB activation. Importantly, the silencing of the LUBAC components is toxic in ABC DLBCL (34, 38), a finding which was further validated in recent genome-wide loss-of-function CRISPR-Cas9 screens (28). Furthermore, two rare polymorphisms in the RNF31 gene encoding for HOIP were found enriched in ABC DLBCL with an overall frequency of 7.8% (38). Such variants increase the interaction between HOIP and HOIL1 and result in an enhanced activity of the LUBAC. Moreover, the introduction in ABC DLBCL cell lines of stapled α-helical peptides to dismantle the LUBAC reduced the aberrant NF-κB activation and was toxic (38).

Although interfering with NF-κB addiction in ABC DLBCL is appealing, its pleiotropic nature may jeopardize the success of its targeting and urges us to establish treatments that selectively kill tumor cells while sparing healthy cells. A clearer understanding of signaling mechanisms and post-translational modifications occurring in ABC DLBCL could provide new potential prognostic markers and putative targets for therapy. In this review, we therefore focus on the post-translational modifications, which can occur within the CBM complex, as this signalosome is instrumental to the survival of ABC DLBCL.

## Phosphorylation of CARMA1 and BCL10 shapes the CBM complex activity

Phosphorylation is considered to be the most prevalent reversible post-translational modification in eukaryotic cells and is crucial for cellular processes including cell cycle, growth, apoptosis and signaling transduction pathways (39). This modification principally occurs on Serine (S), Threonine (T), or Tyrosine (Y) residues to regulate protein/enzyme function by either activating or deactivating them (39). It is therefore not surprising that both CARMA1 and BCL10 are extensively phosphorylated upon antigen receptor engagement (20, 32, 40–55).

### CARMA1 phosphorylation

**Figure 2:**
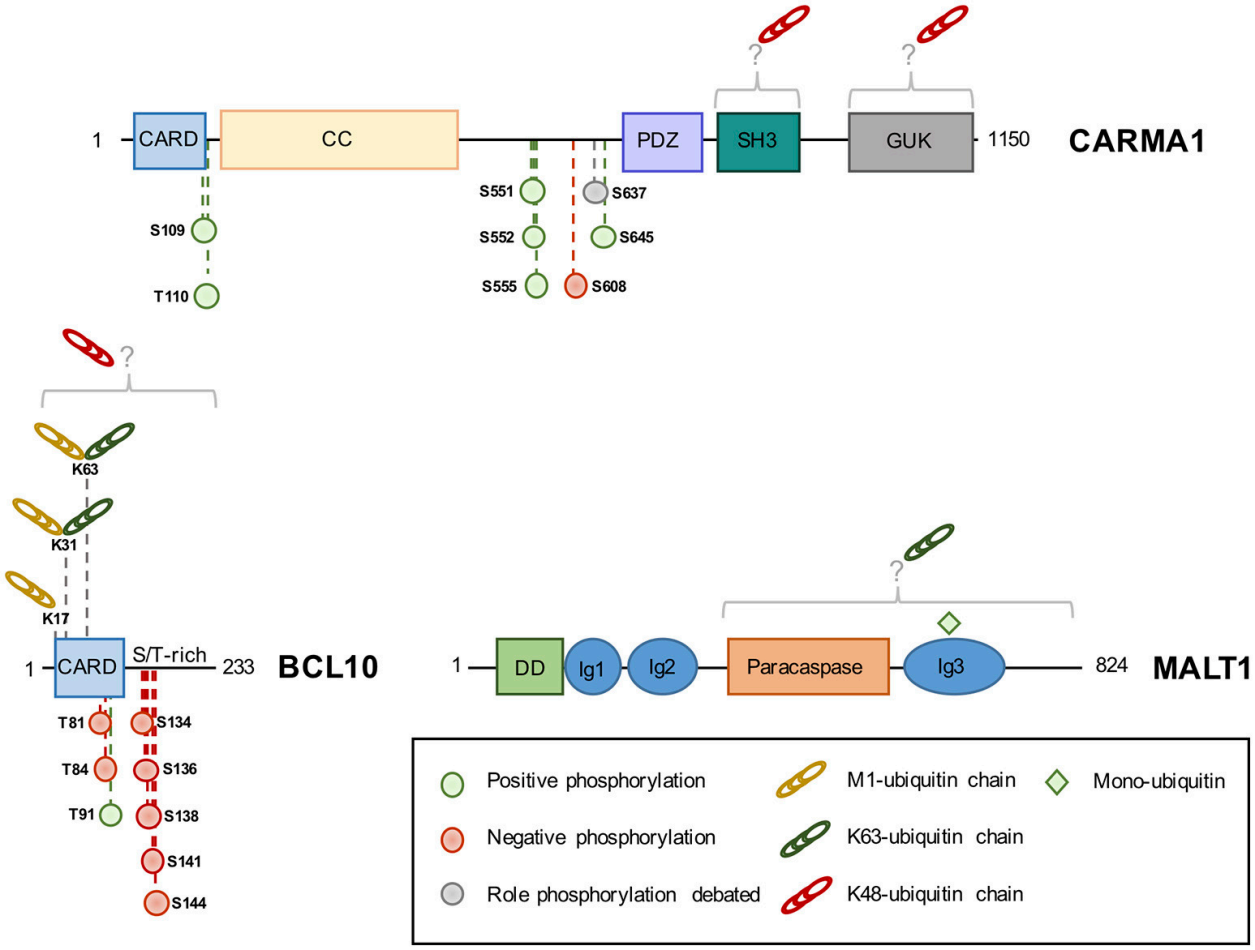
The domain structure of CBM components and their identified phosphorylation and ubiquitination sites. Phosphorylation of CBM components can positively (green circle) or negatively (red circle) influence NF-κB, although the role of some sites is debated (gray circle). K^48^-linked ubiquitin linked (red chains) chains lead to protein degradation, while K^63^ or M^1^-linked chains (green and yellow chains, respectively) modulate activity. Monoubiquitination (green square) activates MALT1 protease activity. CARD, Caspase recruitment domain; CC, Coiled-Coil; PDZ, PSD95-DLG-ZO1 homology domain; SH3, Src homology 3; GUK, guanylate kinase domain; S/T-rich, serine/threonine-rich domain; DD, death domain; Ig, immunoglobulin-like domain; K, lysine; M, methionine.

CARMA1 consists of a caspase recruitment domain (CARD), coiled-coil (CC), PSD95, DLG and ZO1 homology (PDZ), Src homology 3 (SH3) and guanylate kinase (GUK) domain (20). Phosphorylation of key Serine residues in the linker domain between CC and PDZ is a crucial step for the formation of the CBM complex upon antigen receptor engagement (20, 32, 40–42, 44, 56) (Figure 2). PKCβ/θ emerged as the most crucial kinase to phosphorylate CARMA1 in its linker domain on S^552^, and S^645^, causing CARMA1 to switch from an inactive “locked” conformation to an active “open” conformation by relieving the autoinhibition caused by the intramolecular association of the linker region to the CARD motif (32, 40). This conformational change in CARMA1 is crucial to enable the CARD-CARD-dependent recruitment of BCL10 and the subsequent activation of the downstream NF-κB pathway (32, 40–42, 57). The PKCθ-dependent phosphorylation of S^645^ is removed by the Serine/Threonine Protein phosphatases family member phosphatase 2A (PP2A), which constitutively binds CARMA1. By dephosphorylating CARMA1, PP2A finely tunes CARMA1 association with BCL10-MALT1 and therefore prevents excessive NF-κB activation (57).

A large number of kinases, besides PKCβ/θ, have been shown to phosphorylate CARMA1 on a variety of sites, influencing the strength of BCL10-MALT1 recruitment and downstream NF-κB activation (32, 42, 55, 58). This includes protein kinase B (PKB or AKT), which also phosphorylates CARMA1 on S^645^, in addition to PKCβ, upon TCR stimulation (55). IKKβ and hematopoietic progenitor kinases 1 (HPK1) were shown to phosphorylate CARMA1 on S^555^ and S^551^, respectively, leading to an optimal NF-κB activation (32, 42, 58). In contrast, phosphorylation of CARMA1 on S^608^ by casein kinase 1α (CK1α) leads to a diminished NF-κB activation (44, 59). However, this negative role is overwhelmed by the crucial adaptor role of CK1α in bridging the CBM and IKK complexes in antigen receptor-stimulated lymphocytes (44). In ABC DLBCL cell lines, CK1α knockdown halts aberrant NF-κB signaling and is toxic (44). CK1α therefore emerges as a non-oncogenic addiction gene, a gene not involved in the initiation of the transformed phenotype but required for the tumorigenesis (60). In addition to S^608^, it was shown that CARMA1 S^637^ phosphorylation also reduced NF-κB activation in a DT40 chicken B cells model (59). AKT was later identified as the kinase in charge of phosphorylating this site. However, interfering with this modification only modestly influenced NF-κB activation in human T-cells. Rather, the phosphorylation of CARMA1 on S^637^ in combination with S^645^ may positively regulate NF-κB activation (55). Outside the linker domain, BCL10 recruitment and NF-κB activation were shown to be enhanced by phosphorylation of S^109^ by Ca^2+^-calmodulin-dependent kinase II (CaMKII) in T cells, and phosphorylation of T^110^ (T^119^ chicken ortholog) by an unknown kinase in chicken DT40-cells (42, 43).

Of note, gain of function mutations were found in the CC domain of CARMA1, in ~10% ABC DLBCL cases (16, 61). Although oncogenic CARMA1 mutants form cytosolic aggregates and spontaneously induces NF-κB signaling, whether they bypass the need for CARMA1 phosphorylation remains unclear (16, 61). In addition, the status of CARMA1 in ABC DLBCL is yet to be defined.

### BCL10 phosphorylation

Unlike CARMA1, whose phosphorylation mostly results in a positive regulation of the NF-κB pathway (see above), the phosphorylation of BCL10 essentially curbs its activity (45–48, 51–53). BCL10 is constitutively bound to MALT1 and consists of a CARD domain and a Serine/Threonine-rich domain (20) (Figure 2). First hints that BCL10 is a phosphoprotein came with the discovery that BCL10 equine herpesvirus 2 viral homolog v-E10 drives BCL10 hyperphosphorylation and redistribution from the cytosol to the plasma membrane (62). In lymphocytes, Wegener et al. identified five different sites (S^134^, S^136^, S^138^, S^141^, and S^144^) on BCL10, which are phosphorylated by IKKβ upon antigen receptor engagement (47). The recruitment of BCL10 to CARMA1 was shown to be seemingly necessary for its phosphorylation (47). IKKβ phosphorylation of BCL10 does not interfere with CARMA1-BCL10 association in activated T cells, but disrupts BCL10-MALT1 interaction, thereby attenuating NF-κB activation (47). Yet, the ability of IKKβ to phosphorylate BCL10 was rather weak, hinting at the involvement of other kinases (47). Indeed, BCL10 can also be phosphorylated on S^138^ by CaMKII in T cells. However, unlike CaMKII phosphorylation of CARMA1, which leads to and increased recruitment of BCL10 to CARMA1 and higher NF-κB activation, CaMKII phosphorylation of BCL10 on S^138^ recapitulates the results found by Wegener et al. and Ishiguro et al. (47, 48).

The majority of studies agree that BCL10 phosphorylation leads to BCL10 degradation, however, the mechanism and molecular players vary between studies and are a matter of debate (45–48, 51). Zeng et al. reported S^138^ BCL10 phosphorylation to signal for BCL10 degradation in a proteasome-independent manner, while Lobry et al. described IKKβ phosphorylation of BLC10 on T^81^ and T^85^ to lead to a proteasome-dependent degradation of BCL10 (45, 51). Conversely, experiments identifying Ca^2+^-dependent phosphatase Calcineurin as a specific S^138^ BCL10 phosphatase in T cells, did not show BCL10 degradation, nor the dissociation of BCL10 from MALT1 (52, 53). However, they confirm S^138^ BCL10 phosphorylation to be vital in curbing NF-κB activation (52, 53). Inhibition of Calcineurin leads to an increased BCL10 phosphorylation and decreases NF-κB activation, and in T-helper cells, the dephosphorylation of S^138^ by Calcineurin was shown to be essential for NF-κB activation (52, 53). Besides this negative role, BCL10 phosphorylation on S^138^ controls T-cell receptor and Fcγ-induced actin polymerization, in a MALT1- and CARMA1- independent manner (54). Finally, BCL10 phosphorylation can also positively regulate NF-κB signaling. For instance, both BCL10 phosphorylation by CaMKII, on T^91^, and GSK3β, on unmapped phosphorylation sites, promote NF-κB signaling in T cells (49, 50).

In essence, the CBM components CARMA1 and BCL10 are regulated by phosphorylation, and depending on the phosphorylation site and kinase involved; phosphorylation can lead to both positive and negative regulation of the NF-κB pathway.

## Control of the CBM activity by ubiquitination

Ubiquitination is a reversible post-translational modification of Lysine (K) residues in proteins with ubiquitin, which governs nearly all cellular processes in eukaryotes (63). A plethora of polyubiquitin chains exist, as ubiquitin itself is a substrate of ubiquitination at eight different position (M^1^, K^6^, K^11^, K^27^, K^29^, K^33^, K^48^, and K^63^). This variety of ubiquitin linkages was proposed to form a code that leads to different cellular outcomes (63). Ubiquitination is a reversible process counteracted by a family of proteases called deubiquitinating enzymes (DUBs). A large body of literature now supports the idea that ubiquitination shapes the activity of the CBM in lymphocytes and lymphoma (64, 65).

### CARMA1 ubiquitination

The engagement of antigen receptors was shown to promote the K^48^-linked ubiquitination of CARMA1 and its subsequent degradation by the proteasome (66). Although not formally identified, the Lysine acceptors were mapped within the SH3 and GUK domains of CARMA1 (Figure 2). The substitution of the 29 Lysine residues with Arginines enhances both basal and TCR-mediated activation of NF-κB (66). Of note, the SH3 and GUK domains form intra- and intermolecular bounds necessary for CARMA1 recruitment at the immunological synapse, and for the activation of NF-κB both in lymphocytes and in ABC lymphoma (67). Yet, the abundance of CARMA1 was not found overtly decreased in ABC DLBCL (28, 34), suggesting that mechanisms that prevent ubiquitination and degradation may take place. Recently, a comprehensive analysis by quantitative mass spectrometry-based proteomics of the landscape of BCR-mediated ubiquitination revealed that CARMA1 is also subjected to linear ubiquitination (35). Future works are therefore required to elucidate the function of this modification.

### BCL10 ubiquitination

In sharp contrast to CARMA1, the ubiquitination status of BCL10 has received a large amount of attention. Although the exact nature of chains bound to BCL10 continues to be deciphered, it is now clear that a mixture of at least K^48^, K^63^ and M^1^ linkages can decorate the adaptor, leading to different cellular outcomes (Figure 2). BCL10 ubiquitination was initially linked to degradation as a mean of terminating NF-κB activation following antigen receptors ligation, and several hypotheses have been proposed. First, the E3 ligases β-TRCP and c-IAP2 were found to catalyze K^48^-linked ubiquitination of BCL10 following its phosphorylation by IKKβ, thus promoting its proteasomal degradation (45, 68). However, ubiquitinated BCL10 can also be eliminated by the lysosome, either in a NEDD4/Itch-dependent manner (46), or once recognized by the autophagy receptor p62 (69). K^48^-linked ubiquitin chains bound to BCL10 were proposed to be removed by the pleiotropic DUB USP9X in lymphocytes (70). Rather than preventing BCL10 degradation, the trimming of K^48^ chains would allow the CBM complex to assemble properly (70). However, the subsequent generation of mice knockout for USP9X in T or B cell compartments revealed a role for USP9X in the CBM restrained to B cells, possibly by selectively regulating PKCβ upstream of BCL10 (71, 72). BCL10 ubiquitination and stability is also under the control of the COP9 signalosome (CSN), a multiprotein complex of the ubiquitin-proteasome system, which participates to the CBM upon TCR engagement (73). Finally, the E3 ligase RNF181 adjusts the basal abundance of BCL10 and therefore, negatively regulates NF-κB signaling in lymphocytes and ABC lymphoma (74). Nevertheless, whether K^48^-linked ubiquitination operates in ABC DLBCL remains to be explored.

Pioneer work by Wu and Ashwell revealed that the binding of K^63^ chains to BCL10 precedes K^48^-linked ubiquitination in lymphocytes (15). In ABC DLBCL, experiments with tandem ubiquitin-binding entities (TUBE) to selectively enrich for polyubiquitinated proteins also showed that BCL10 is merely attached to K^63^-linked chains (75). This non-degradative ubiquitination requires an intact CBM complex and was proposed to recruit the LUBAC complex and allow IKK activation (15, 34, 36, 75, 76). Mutagenesis experiments identified the Lysine residues K^31^ and K^63^ as the main acceptors of ubiquitin in TCR-stimulated Jurkat cells as well as in ABC DLBCL (15, 36, 75, 76). Their substitution to Arginine hampered TCR-mediated NF-κB activation (15, 75). Yang et al. elegantly demonstrated that the E3 ligases cIAP1/2 were integral to the CBM complex and promoted BCL10 K^63^-linked ubiquitination in ABC DLBCL (75). The treatment of ABC DLBCL with the bivalent SMAC mimetic Birinapant to eliminate cIAP1/2 was toxic, as it disrupted the constitutive CBM-LUBAC signalosome and hampered aberrant NF-κB activation (75). Moreover, Birinapant inhibited tumor growth *in vivo* in BCR-dependent ABC DLBCL xenografts models (75). Of note, the requirement for c-IAP1/2 may be more restricted to chronic or tonic stimulation of the BCR, as no overt defect in TCR-mediated NF-κB activation was observed in Birinapant-treated Jurkat T cells (our unpublished observations).

The discovery that the LUBAC binds to the CBM in lymphocytes and lymphoma suggested the existence of substrate(s) within this signalosome (34, 38). However, whether catalytic activity of the LUBAC is crucial was initially disputed, as NF-κB signaling can occur in antigen receptors-stimulated dead-HOIP cells and was not significantly altered by the silencing of OTULIN (34, 77). Nevertheless, a combination of TUBE, SILAC, and standard biochemistry revealed that BCR and oncogenic BCR stimulation led to linear ubiquitination of BCL10 (35, 75). In addition, the overexpression of oncogenic CARD11 mutations also drove the LUBAC to decorate BCL10 with M^1^-linked chains (36). Supporting a positive role for this linkage, a fusion protein of BCL10 conjugated to four linearly linked DUB-resistant ubiquitin moieties enhanced the activation of NF-κB when ectopically expressed (35). In Jurkat T cells, the ligation of TCR also leads to the conjugation of M^1^ chains on BCL10, which required the Lysine residues 17, 31, and 63 (36). Because K^31^ and K^63^ are also targeted for K^63^-linked ubiquitination (15), it is tempting to speculate that K^63^/M^1^ branched poly-ubiquitin chains bind BCL10 (78). In keeping with this idea, BCL10 K17R mutation was sufficient to impair linear and K^63^-linked ubiquitination of BCL10 and to dampen TCR-mediated NF-κB activation (36).

### MALT1 ubiquitination

MALT1 K^63^-linked poly-ubiquitination has been observed in lymphocytes shortly following antigen receptor stimulation, as well as, in ABC DLBCL (14, 34, 44, 73, 79, 80). In their seminal work, Oeckinghaus et al. identified 11 putative Lysine acceptors in the COOH-terminal segment of the protein, which abolished ubiquitination and recruitment of IKK when mutated to Arginines (14). MALT1^−/−^ CD4^+^ T lymphocytes reconstituted with a ubiquitin-resistant MALT1 mutant display reduced NF-κB activation and diminished production of IL-2 (14). Several lines of evidence have linked the E3 ligase TRAF6 to MALT1 ubiquitination. First, TRAF6 can directly binds the COOH-terminal part of MALT1 via two consensus binding sites (81, 82). In addition, oligomers composed of BCL10 and MALT1 promote the binding and activation of TRAF6 enzyme activity (81). Moreover, siRNA-mediated silencing of TRAF6 in Jurkat cells abolished MALT1 ubiquitination and dampened TCR-mediated NF-κB activation (14, 83). However, T-cell-specific deletion of TRAF6 had no overt effect on TCR-mediated activation of NF-κB, IL-2 production or proliferation (84). Of note, the abundance of TRAF6 is rather low in naïve mouse CD4^+^ T cells and is rapidly upregulated following the engagement of antigen receptors, suggesting that TRAF6 may be more relevant in activated lymphocytes (84). It is also possible that TRAF6 action is redundant with additional E3 ligases, such as TRAF2 (81). Shortly following TCR stimulation in Jurkat cells, the DUB A20 is recruited to the CBM (our unpublished results), to selectively remove K^63^-linked ubiquitin attached to MALT1 and therefore ensuring optimal recruitment and activation of the IKK complex (79). In return, A20 is rapidly cleaved and inactivated by MALT1 (see below). Whether MALT1 poly-ubiquitination participates in its paracaspase activity is unknown. Interestingly, the deubiquination of MALT1 still occurred without A20, although delayed, suggesting that other DUBs may participate to this process (79). MALT1 is also monoubiquitinated on the Lysine residue 644 shortly after TCR stimulation in Jurkat, or in a constitutive manner in ABC DLBCL cell lines (Figure 2) (85). This monoubiquitination is driven by the dimerization of MALT1 and requires a functional CBM complex (85, 86). A monoubiquitination-resistant mutant was not able to completely unleash the protease activity of MALT1 (85–87). MALT1 K644R-expressing cells displayed reduced cleavage of MALT1 substrates and diminished NF-κB activation (85, 86). In ABC DLBCL, interfering with MALT1 monoubiquitination was toxic (85).

## MALT1 protease activity tunes the immune response and is required for lymphomagenesis

Although MALT1 encompasses a COOH-terminal paracaspase domain that shares homology with caspases, it was initially thought to lack proteolytic activity and to only serve as a scaffold adaptor (88). In 2008 however, Margot Thome's and Rudi Beyaert's laboratories established MALT1 as a functional protease that cleaves substrates after an arginine residue embedded in a consensus S/P-R↓G/A site (89, 90). As previously mentioned, the activation of MALT1 results from a monoubiquitination on the Lysine residue 644 upon antigen receptor engagement (85). Recently, the generation of mice expressing a catalytically dead MALT1 uncovered the essential role of the protease in lymphocyte development and activation (91–94). Notably, MALT1 protease activity was demonstrated to control the development of regulatory T cells (T_Reg_) and T helper 17 (T_H_17) cells, as well as, innate-like B cells. Furthermore, protease dead mature T lymphocytes displayed proliferation impairment and reduced TNFα and IL-2 secretion (91–94). Interestingly, MALT1 inactivation protected the mice against experimental autoimmune encephalomyelitis (EAE) and colitis, suggesting a critical role for MALT1 in the control of autoimmune diseases (91–94). Nevertheless, the deletion of MALT1 proteolytic activity is also deleterious, as mice expressing a catalytically dead MALT1 develop a lethal multi-organ inflammatory syndrome, arising from aberrant secretion of interferon gamma (IFNγ) by effector lymphocytes (91). Interestingly, several specific types of tissue damage have been reported upon protease inactivation ranging from neurodegeneration (91), gastric inflammation (91–94), lung immune infiltration (94) to eye inflammation (93). Furthermore, proteolytic dead MALT1 mice developed signs of autoimmune gastritis associated with high serum levels of IgE and IgG1 resulting from accumulation of activated T cells and a loss of T_Reg_ cells (92). In keeping with this, reconstitution of MALT1 protease dead mice with T_Reg_ overcame this defect and delayed the multi-organ inflammatory pathology and lethality (93). Strikingly, MALT1^−/−^ mice lacking the paracaspase are immunodeficient but do not display any overt pathology (95, 96). In addition, several human patients with MALT1 deficiency have been reported to suffer from combined immunodeficiency disorders (CID) (97, 98). The differences observed in MALT1 deficiency and protease inactivation support a balanced function of MALT1 as a scaffold and as a protease cleaving a subset of substrates.

### MALT1 cleaves regulators of NF-κB activation

Over the last decade, our understanding of the landscape of MALT1 functions has improved with the discovery of ten substrates (99) (Table 1). Among them, a subset of substrates (A20, RelB, HOIL1, MALT1, and NIK) participates in the regulation of NF-κB signaling. As previously discussed, the DUB A20 negatively regulates NF-κB activation in response to various stimuli by specifically removing K^63^ ubiquitin chains from key signaling adaptors, such as TRAF6, NEMO, and MALT1 (89, 115). Upon TCR engagement, MALT1-mediated cleavage of A20 at the R^439^ residue promotes proteasomal degradation of the resulting fragments, and limits its negative action on NF-κB signaling (79, 89). However, no overt change in IKK-dependent phosphorylation of IκBα was observed in the absence of MALT1 activity (92). Further work is therefore needed to precisely pinpoint the effect of A20 processing, and to determine if the generated fragments could have a role on NF-κB activation independently of IKK. Interestingly, A20 has been shown to function as a tumor suppressor in multiple cancer like mantle cell lymphoma (MCL) (116), mucosa-associated lymphoid tissue (MALT) lymphoma (117) and in DLBCL (118). Because this DUB presents inactivating mutations in several hematological malignancies, studying the status and role of A20 processing could reveal some insight into the molecular mechanisms at hand and offer relevant therapeutic strategies.

**Table 1:** MALT1 substrates, cleavage sites and their influence on cellular function.

**Substrate**	**Cleavage site**	**Function**	**References**
A20	GAS**R**^439^GEA	NF-κB activation	(89, 98)
RelB	LVS**R**^85^GAA	NF-κB activation	(100)
HOIL1	LQP**R**^165^GPL	NF-κB activation	(101–103)
MALT1	LCC**R**^149^ATG… HCS**R**^781^TPD	NF-κB activation	(104–107)
NIK	CLS**R**^325^GAH	NF-κB activation	(108)
LIMA1	PDS**R**^206^ASS…FKS**K**^289^GNY	B cell growth, adhesion	(109)
CYLD	FMS**R**^324^GVG	JNK/AP-1	(110)
Roquin 1	LIP**R**^510^GTD…MVP**R**^579^GSQ	mRNA stability	(111)
Roquin 2	LIS**R**^509^TDS	mRNA stability	(111)
Regnase	LVP**R**^111^GGS	mRNA stability	(111, 112)
BCL10	LRS**R**^228^TVS	Adhesion	(90)
Unknown		mTOR	(113, 114)

Another MALT1 substrate involved in NF-κB signaling is the NF-κB family member RelB (100). The NF-κB transcription family is comprised of five members that all share a Rel homology domain (RHD) required for oligomerization and DNA binding. NF-κB activation can rise from the canonical pathway that relies on the formation of RelA-p50 or c-Rel-p50 complexes or the non-canonical pathway that leads to the formation of RelB-p50 heterodimers. RelB was proposed to negatively regulate canonical NF-κB activation by competing for DNA binding sites, as well as, by forming transcriptionally inactive complexes with RelA and c-Rel (100). RelB processing upon antigen receptor engagement in Raji B cells, lymphoblastoid Jurkat, and in human primary CD4^+^ T cells is followed by rapid proteasomal degradation of the produced fragment. The ectopic expression of a MALT1-resistant RelB mutant led to a decrease in NF-κB activation assessed by gene reporter assay in Jurkat, while RelB silencing increased IL-2 production in naïve primary T cells (100).

Three independent groups including ours, recently identified the LUBAC subunit HOIL1 as a substrate of MALT1 (101–103). Although the LUBAC has been shown to be an integral part of the CBM required for IKK activation in lymphocytes and in ABC DLBCL (34, 38), the role of HOIL1 has remained rather elusive. Our *in-silico* analysis of the known CBM partners led to the identification of HOIL1, as a putative MALT1 substrate (103). Experiments with Jurkat cells, human primary T lymphocytes and mouse primary T lymphocytes further validated that HOIL1 was processed by MALT1 at R^165^ upon antigen receptor engagement (102, 103). Of note, API2-MALT1 fusion oncoprotein resulting from the recurrent t(11;18)(q21;q21) in MALT lymphoma also hydrolyses HOIL1 at R^165^ (102). Concurrently, Klein et al. also identified HOIL1 as a substrate of the paracaspase by studying the B cells profile in a MALT1 deficient patient using 10-plex Tandem Tag TAILS N-Terminal peptide proteomics (101). The authors further propose that HOIL1 processing destabilizes the LUBAC, impairs linear ubiquitination, therefore reducing NF-κB signaling (101). However, we favor a model, in which MALT1 cleaves and alleviates HOIL1 negative regulation on NF-κB (103). Indeed, the expression of a MALT1-resistant HOIL1 mutant in Jurkat cells hampered NF-κB activation and subsequent IL-2 secretion in response to the ligation of TCR (103). This suggests that HOIL1, together with A20 and RelB, belongs to a subset of MALT1 substrates that negatively regulate NF-κB when left uncleaved. Nevertheless, further work needs to be done to precise the function of the fragments generated by HOIL1 processing.

Interestingly, recent work report that MALT1 also contributes to full NF-κB activation via its auto-proteolysis at R^149^ (104). Expression of a non-cleavable MALT1 mutant led to a decrease in IL-2 production in Jurkat T cells without affecting phosphorylation of IκBα or cleavage of other MALT1 substrates. Furthermore, a qRT-PCR approach revealed that MALT1 auto-processing is essential for expression of NF-κB target genes in activated Jurkat cells (104). Another study confirmed the function of MALT1 self-cleavage in driving IL-2 production in CD4^+^ T cells, and showed a subsequent function in controlling T_Reg_ homeostasis. Strikingly, overexpression of a MALT1 unable to self-process led to a defect in T_Reg_ number and an increase in anti-tumoral immunity reactivity (105). More recently, MALT1 was reported to also auto-cleave at R^781^ upon TCR engagement. Overexpression of a processing resistant mutant impaired NF-κB activation and consequent IL-2 production (106). Recently, TRAF6 was shown to induce MALT1 auto-proteolysis at R^149^ generating a fragment with high signaling properties whereas processing at R^781^ was shown to abrogate TRAF6 binding and subsequent NF-κB activation (107). Of note, MALT1 seems to control the non-canonical NF-κB signaling pathway as well, by processing the NF-κB induced kinase (NIK) in the context of the oncogenic fusion protein API2-MALT1 (108). NIK phosphorylates NF-κB p100 subunit to trigger its processing into the active p52 subunit (119). This pathway is tightly regulated by the short lifespan of NIK that is rapidly degraded by the proteasome. Strikingly, API2-MALT1 cleaves NIK at the R^325^ to generate a fragment containing the kinase activity and resistant to degradation. The subsequent NF-κB activation was shown to drive B cell adhesion and resistance to apoptosis (108). Importantly, MALT1 only cleaves NIK independently of the CBM complex in the context of API2-MALT1. This is also the case of the tumor suppressor LIM domain and actin-binding protein 1 (LIMA1), which promotes B-cell growth, adhesion and tumorigenicity when cleaved (109).

### MALT1 processes the deubiquitinating enzyme CYLD

In addition to its role in finely tuning NF-κB, MALT1 was also linked to JNK/AP-1 signaling. Purified T cells from MALT1-deficient mice displayed impairment in JNK activation upon antigen receptor engagement (95). Staal et al. showed that T cell receptor ligation as well as overexpression of the oncogenic API2-MALT1 fusion proteins led to the processing and inactivation of CYLD at R^324^ (110). CYLD is a deubiquitinating enzyme that acts as a negative regulator of the JNK and AP-1 pathways by removing ubiquitin from TAK1 (120). The overexpression of a MALT1-resistant CYLD mutant led to a decreased expression of JNK and AP-1 target genes like IL-2, IL-8, and c-Jun (110). Yet, siRNA-mediated silencing of the DUB only partially increased JNK signaling, suggesting other DUBs could be involved. Moreover, mice expressing a catalytically dead MALT1 do not present overt defects in JNK and AP-1 signaling, suggesting additional functions for CYLD processing (93).

### MALT1 promotes mRNA editing

Another unexpected function of MALT1 was further characterized with the discovery that Regnase-1 and Roquin1/2, proteins involved in RNA stability, are substrates of the paracaspase (111, 112). Regnase-1 is an RNase that mediates the mRNA stability of several genes including c-Rel, OX40, and IL-2 and is essential for the prevention of aberrant CD4^+^ effectors T cells generation (112). In keeping with this, mice deficient for Regnase-1 develop severe systemic inflammation resulting from B and T cell activation (121). Uehala and colleagues reported that antigen receptor engagement induced the cleavage of Regnase-1 at R^111^ by MALT1 and subsequent proteasomal degradation of the generated fragment. Interfering with MALT1 protease activity led to the destabilization of a subset of mRNA and therefore to a deregulation in T cell activation (112). In addition to its role in CD4^+^ T effector differentiation, Regnase-1 has been shown to act together with the RNA binding proteins Roquin1 and Roquin2 to control T_H_17 effector differentiation and IL17 production (111). Roquin 1 and 2 were also demonstrated to be processed by MALT1 following antigen receptor signaling. Roquin deficient T cells have upregulated IκBζ and IκBNS transcripts, driving aberrant T_H_17 differentiation. This phenotype was reversed by expression of a MALT1-insensitive Roquin mutant (111).

### MALT1, adhesion, and metabolic signaling

The spectrum of MALT1's function was widened with the discovery of its involvement in adhesion, and metabolic signaling via mTORC1. Upon TCR engagement, MALT1 cleaves its binding partner BCL10 at R^228^ to ensure optimal adhesion of cells to fibronectin, through mechanisms that remain poorly understood (90). In 2014, Nayaka and colleagues reported that the CBM complex and MALT1 paracaspase activity are required for glutamine uptake and mTORC1 activation upon TCR engagement, independent of IKK (113). The use of z-VRPR.fmk led to a decrease in glutamine uptake in adition to phosphorylation of mTORC1 targets S6 and S6K1. The role of MALT1's protease activity in driving metabolic flux was concurrently reported in activated CD4^+^ T cells (114). In keeping with this, a recent work identified 4 rare hypomorphic dominant negative mutations in CARMA1 that interfere with the activation of MALT1 and mTORC1 (122). Conversely, mTORC1 signaling is increased in a B-cell intrinsinc expression of an activating mutation in CARMA1 model (123). How MALT1 enzyme links antigen receptors to mTORC1 activation and metabolic changes remains unclear, as the substrates involved are yet to be identified.

### MALT1 protease and ABC DLBCL

While dormant in resting lymphocytes unless stimulated through their antigen receptors, MALT1 protease is constitutively active in ABC DLBCL and contributes to pathogenesis (124, 125). Pioneer works by Margot Thome and Jurgen Ruland laboratories reported that inhibition of the catalytic activity of the paracaspase with the tetrapeptide inhibitor z-VRPR.fmk and overexpression of a catalytically dead MALT1 specifically decreased growth and survival of ABC DLBCL lines (124, 125). These results opened the path to several additional studies both *in vitro* and *in vivo*, providing a rationale for targeting the paracaspase in these types of lymphomas. Notably, additional molecules, such as the small compound MI2 or phenothiazines were shown to directly bind and suppress MALT1 protease activity, and were reported to be selectively toxic to ABC DLBCL cell lines *in vitro* as well as xenotransplanted ABC DLBCL *in vivo* without displaying toxicity in mice (111, 126). Nevertheless, the role of MALT1 substrates in ABC DLBCL survival remains poorly characterized. For instance, BCL10, A20, RelB, CYLD, HOIL1 and MALT1 itself were found constitutively processed in a panel of ABC DLBCL cell lines, and only the impact of RelB and MALT1 (R^781^) were extensively assessed. Indeed, Hailfinger et al. conclusively demonstrated that the overexpression of RelB was toxic in ABC DLBCL cell lines (85). Wu et al. showed that overexpression of a non-self-cleavable MALT1 (R^781^) mutant impaired cell viability in HBL-1 (106). Surprisingly, MALT1 substrates, such as Roquin1/2 and Regnase have not been studied in the context of ABC DLBCL and whether MALT1 aberrant activation modulates the stability of mRNAs in this subtype of lymphoma is unknown. Moreover, the exact roles of the tumor suppressors A20 and CYLD in ABC DLBCL remain elusive. Identifying if some substrates are differentially cleaved in ABC DLBCL and precising the function of MALT1 processing seems essential to better understand the pathology which could uncover relevant therapeutic targets. Hence, additional work is necessary to provide a comprehensive understanding of the precise role of each substrate in the pathology.

## Concluding remarks

Although in recent years we have witnessed a tremendous amount of progress in our understanding of the post-translational modifications of the CBM complex upon antigen receptor stimulation, key questions remain about their status and functions in ABC DLBCL: Are CARMA1 and BCL10 phosphorylated in ABC lymphoma? What, if anything, is the impact of their inhibition? In that view, the clinically available PKC inhibitor Sotrastaurin led to a diminished NF-κB activation and offered a significant anti-tumor effect in a subcutaneous CD79-mutated ABC DLBCL xenograft model (30). In keeping with this idea, interfering with the ubiquitination of the CBM subunits may be another attractive path to pursue. Indeed, the cIAP inhibitor Birinapant, which prevents K^63^-linked ubiquitination of BCL10 impedes tumor growth in ABC DLBCL xenograft models (75). One other approach may lie in the generation of small-molecule inhibitors highly specific of deubiquitinating enzymes, as recently demonstrated for USP7 (127, 128). Finally, strategies targeting the MALT1 enzyme in ABC DLBCL have shown promising results. However, genetic paracaspase dead mouse models suggests that systematically blocking MALT1 activity may be harmful. A better understanding of the landscape of MALT1 substrates and their specific roles is therefore required. Also, are all substrates cleaved in ABC DLBCL? The development of technologies, such as genome editing with CRISPR/Cas9, will be helpful to generate MALT1-resistant mutants, and therefore to pinpoint the exact function of each individual substrate (129–131).

Dysregulation in the activity of the CBM subunits is not restricted to ABC DLBCL, and has been linked to T-cell and B-cell lymphoma, cancer, lymphoproliferation, allergy, inflammatory diseases, and primary immunodeficiencies [for review, see (132)]. Greater understanding of the functions of post-translational modifications of the CBM in ABC DLBCL will be essential to develop new diagnostic, prognostic and therapeutic strategies.

## Author contributions

AT, TD, and NB designed and wrote the review.

### Conflict of interest statement

The authors declare that the research was conducted in the absence of any commercial or financial relationships that could be construed as a potential conflict of interest.
